# Health Risks of Kretek Cigarettes: A Systematic Review

**DOI:** 10.1093/ntr/ntab016

**Published:** 2021-01-27

**Authors:** Desy Nuryunarsih, Sarah Lewis, Tessa Langley

**Affiliations:** 1 Division of Epidemiology and Public Health, University of Nottingham, Nottingham, UK; 2 SPECTRUM Consortium, Nottingham, UK

## Abstract

**Introduction:**

The objective of this study is review the evidence on the health risks associated with smoking kretek cigarettes compared with not smoking or smoking regular cigarettes.

**Methods:**

We conducted a systematic literature search in five electronic databases: EMBASE (Ovid), ASSIA, PubMed and Scopus. Since kretek use is largely restricted to Indonesia, we identified additional studies using an online search for grey literature and studies in Indonesian journals and the National Library of Indonesia. We included relevant search terms in English (“kretek” and “clove cigarettes”) and Bahasa (“rokok” and “merokok”). We selected studies which compared any health outcome between smokers of kretek cigarettes and non-smokers or smokers of regular cigarettes. We included studies in any smokers compared to non-smokers in Indonesia, since most Indonesian smokers use kretek, but analysed these separately. Study data were extracted by a single reviewer and checked by two reviewers. Methodological quality was assessed using the Newcastle-Ottawa scale.

**Results:**

We identified 32 studies, all from Indonesia. There were 31 cross-sectional studies and one case control study. This systematic review identified a relatively limited number of studies, and most of these were of poor quality as assessed by the Newcastle Ottawa score. They were generally cross-sectional, small and lacking justification for sample size, had high potential for selection bias because of lack of data on non-respondents or those lost to follow up, and missing information about the statistical analysis. Fourteen studies looked specifically at kretek exposure and eighteen looked at any type of cigarette exposure but were conducted in Indonesia are therefore likely to predominantly reflect kretek exposure. Kretek were found to be associated with oral cancer, cardiovascular disease, chronic health disease, myocardial infarction, asthma, and oral diseases.

**Conclusions:**

Although existing studies are of poor quality, kretek are likely to be at least as harmful as regular cigarettes.

## Introduction

The health effects of manufactured and hand-rolled cigarettes are well established.^1^ In Indonesia, where the current prevalence of tobacco use is 4.8% among women and 62.9% among men,^2^ clove cigarettes, known as kreteks, are the most popular tobacco product, making up almost 90% of all cigarette sales.^3^ In recent years, the transnational tobacco companies have entered the kretek market in Indonesia,^4^ and the Indonesian government has yet to ratify the Framework Convention on Tobacco Control.^5^

The global prevalence of kretek use is low, and there is therefore relatively limited research on this tobacco product. However, kreteks have been found to yield higher levels of tar and carbon monoxide than regular cigarettes.^6^ Although clove buds contribute to high levels of eugenol in kretek smoke, the “sauce” used to flavor kreteks may also contain harmful ingredients.^7^

Given the constituents of kretek cigarettes, there is no scientific basis for concluding that a kretek is any less hazardous than a regular cigarette.^8^ Thus the health effects of kretek cigarettes are likely to be at least as detrimental as those of regular cigarettes. However, the evidence has not been systematically reviewed. We have systematically synthesized and reviewed the evidence from studies which have compared the health risks of kretek smoking compared with not smoking or regular cigarettes.

## Methods

The PRISMA (Preferred Reporting Items for Systematic Review and Meta-Analysis) guidelines were used to conduct this systematic review.^9^ The study protocol was published in PROSPERO (registration number CRD42017079757).

### Inclusion Criteria

Throughout the review process, we used PECOS (Population-Exposure-Comparison-Outcomes-Study design) to assess the eligibility of the identified studies shown in Supplementary Appendix 1. We included any human population around the world. We included all studies assessing kretek cigarette consumption, people who smoke kretek cigarettes and people exposed to kretek cigarettes (involuntarily inhaling kretek cigarette smoke). We included studies comparing current kretek smokers with nonsmokers, current kretek smokers with regular cigarette smokers, nonsmokers passively exposed to kretek cigarettes alongside unexposed nonsmokers, or nonsmokers exposed to regular cigarettes. The outcomes of interest were the short-term and long-term health effects of smoking kretek cigarettes or exposure to kretek cigarettes, as well as any benefits to health from the use of kretek cigarettes (if any). Any physical or mental health-related outcome was included. We included (if any) existing systematic review studies, comparative longitudinal studies, or cohort studies, assessing the associated health risk of kretek cigarettes. Case–control and cross-sectional studies were also included.

Most cigarettes consumed in Indonesia are kretek cigarettes^10^; thus, we included any studies on the health effects of smoking in Indonesia. Studies conducted in Indonesia that did not specifically mention whether the exposure was kretek cigarettes (as opposed to regular cigarettes) were analyzed separately. There was no restriction on the publication period or language; however, only relevant studies in Bahasa Indonesia and English were identified.

### Search Strategy and Study Selection

We conducted a systematic literature search using a search strategy, which was developed with the support of an expert librarian. We conducted searches to include all materials up to and including September 2017.

We searched five major electronic databases: EMBASE (Ovid), ASSIA (Applied Social Science Index and Abstracts), PubMed, and Scopus. To identify additional, geographically relevant information (ie, Indonesian studies), we conducted an online search for gray literature and searched Indonesian medicine and public health journals (from 82 universities in Indonesia), and the National Library of Indonesia. We included relevant search terms in English (“kretek” and “clove cigarettes”) and since kretek use is largely restricted to Indonesia, in Bahasa (“rokok” and “merokok”). The full search strategy is presented in Supplementary Appendix 2.

Double screening of a list of deduplicated titles and abstracts was conducted independently by DN and TL. The full texts of potentially eligible articles were identified and read independently by two reviewers (TL and DN) to check if they were in line with the inclusion criteria. Disagreements were resolved through discussion with a third reviewer (SL). For the articles in Bahasa Indonesia, full texts of potentially eligible articles were identified and read by (DN) and discussed in English with TL and SL.

### Data Extraction and Quality Assessment

Study data were extracted by a single reviewer (DN) and were checked by TL and SL. Three main categories of data were extracted: the methodological characteristics of each study, characteristics of the samples, and estimated effects of kretek cigarette exposure on health outcomes. We subsequently undertook narrative synthesis of the included studies.

The assessment of the methodological quality of the primary studies was conducted using the Newcastle–Ottawa scale.^11^ All of the included studies were cross-sectional or case–control studies. The Newcastle–Ottawa scale evaluates three quality parameters (selection, comparability, and outcome)^9,12^ divided across eight specific items, which differ slightly for the scoring of case–control and longitudinal studies.^12^

In the Newcastle–Ottawa scale assessment for case–control studies, a study can be awarded a maximum of nine stars. Three researchers (DN, TL, and SL) independently assessed the quality of the studies. Disagreement between the researchers was resolved by discussion. For articles in Bahasa, Indonesia, DN described the studies in English to SL and TL, and together they assessed the quality of the studies.

### Selection Data for Meta-analysis

We intended to undertake meta-analysis if the included studies had sufficiently similar exposures and outcomes. Review of retrieved papers studies were too heterogeneous to allow meta-analysis.

## Results

### Overview of Included Studies

**Figure 1.**
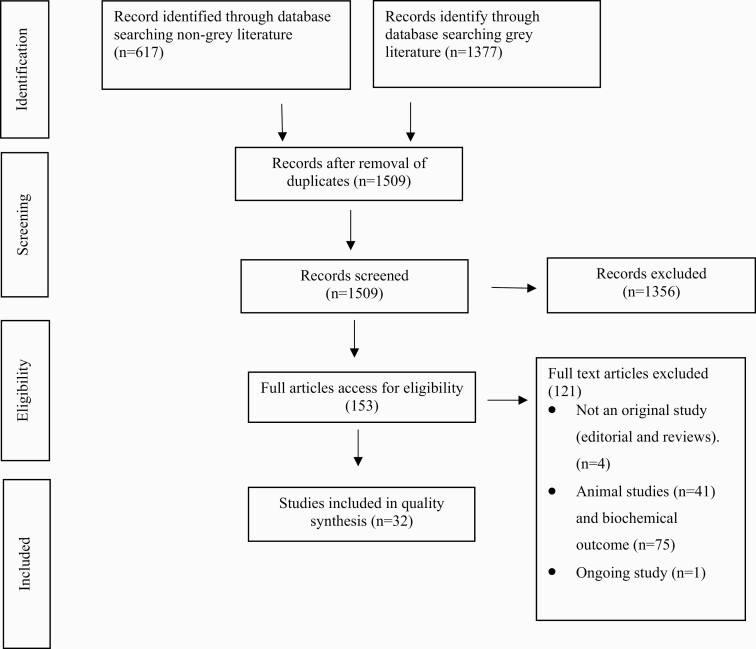
PRISMA diagram of study selection.

Our literature search identified 1509 articles; following title and abstract screening, we reviewed the full texts of 153 that were potentially relevant. Thirty-two studies met the inclusion criteria and were included in the narrative synthesis. Fourteen studies looked specifically at kretek exposure, and 18 studies looked at any type of cigarette exposure but were conducted in Indonesia are therefore likely to predominantly reflect kretek exposure. Figure 1 summarizes the process of study selection.

Studies looking specifically at the association between active kretek cigarette exposure and health risks looked at the following outcomes: oral cancer,^13^ cardiovascular disease,^14^ coronary heart disease,^15^ myocardial infarction,^16^ asthma,^17^ dental decay,^18^ taste sensitivity,^19^ stomatitis nicotine,^20^ periodontal disease,^21^ coated tongue,^22^ oral mucosal lesion,^23^ waist–hip ratio,^24^ and hyperpigmentation in the face.^25^ One study looked at the association between passive kretek exposure and gingival melanin pigmentation.^26^

Studies investigating the association between unspecified cigarette (ie, not specifically kretek) exposure and health outcomes looked at hypertension,^27–31^ coronary heart disease,^32^ forced expiratory flow 25–75%,^33^ cardiorespiratory endurance,^34,35^ asthma,^36^ lung tuberculosis,^37^ depression,^25^ insomnia,^38^ obesity,^39^ eye cataract,^40^ Parkinson’s disease,^41^ and oral gingivitis.^42^

### Study Characteristics

The characteristics of the 32 studies included in this systematic review are shown in Supplementary Table 1. There were 31 cross-sectional studies and one case–control study. All included studies were conducted in Indonesia. Seventeen studies included only men, no study included only women and one study included children. Studies were conducted in different populations: patients in hospital (eight studies)^13,15,16,26,30,32,36,41^; students in universities/schools (eight studies)^22,25,34,35,38,39,42,43^; national surveys (four studies)^14,17,37,44^; drivers—bus^18^ and pedicab^19^; local populations/villages (six studies)^23,27–29,31,33^; farmers^40^; miners^20^; and fisherman.^24^ One study did not describe the population.^21^

The measurement of outcomes varied, and included diagnosis by the researchers, medical records, and interviews. Smoking status was mostly ascertained through self-reporting; one did not mention the ascertainment method.

There were two studies that compared kretek with not smoking and other types of cigarettes^13,14^; one study compared kretek smoking and not smoking^19^; six studies compared kretek with regular cigarettes^15,20–23,43^; 10 studies compared unspecified type of cigarette and not smoking^27,30,32,33,35,37–39,41,44^; one study compared filter and not smoking and nonfilter^29^; six studies investigated unspecified type of cigarette without comparator^25,28,31,34,36,42^; one study compared unspecified type of cigarette and not smoking and ex-smokers^40^; two studies compared kretek with kretek based on level of consumption and nicotine content^16,18^; and one study investigated effect of passive smoking.^26^ Five studies adjusted for a variety of confounders (location, age, gender, educational status, etc.).^13,14,16,26,44^

### Quality Assessment

In this systematic review, none of the included studies were found to be of high quality (Table 1). The main reasons for lower scores in the risk of bias assessment were potential for selection bias because of lack of data on nonrespondents or those lost to follow up and lack of justification of sample size.

**Table 1.** Summary of Quality Assessment for the Included Studies

NOS case control	Case definition	Representative	Selection of controls	Definition of controls	Comparability	Ascertainment of exposure	Same method of ascertainment of case and controls	Nonresponse rate	Score /9
Amtha	*	*	*		*	*		*	6
NOS cross-sectional	Representativeness of the sample	Sample	Nonresponse	Ascertainment of exposure	Comparability	Assessment of the outcome	Statistical test		Score /10
Wasis		*		*	**	*	*		6
Ratnawulan				*			*		2
Wagiu				*	*	*	*		4
Suharmiati	*			*	**	*	*		6
Soetiarto	*			*		*	*		4
Simamora						**	*		3
Siwi	*			*		**			4
Nelis				*		**	*		4
Kaur				*		**	*		3
Djokja	*	*		*		*			4
Khaira				*		**	*		4
Prasetya				*		**	*		4
Setiadhi				*		**	*		4
Yashinta	*			**		**	*		6
Untari	*			**		**	*		6
Narayana				*		**			3
Santosa				*		**	*		4
Hikmah				*		**	*		3
Ramandika				*		*	*		3
Erawati				*		**	*		4
Sukmawati				*		**	*		4
Rizaldy				*		**	*		4
Putra				*		**	*		4
Ernawati	*			*		*	*		4
Wibowo				*		**	*		4
Peng	*	*		*	*	*	*		6
Annahri				*		*	*		3
Lestari				*		**	*		4
Tana	*	*		*		**	*		6
Noviani				*		**	*		4
Syawal				*		**			3

### Summary of Findings on the Health Effects of Kretek

In this systematic review, several studies found a significant association between smoking kretek cigarettes and several health risks: cancer,^13^ disease of circulatory system and blood respiratory disease,^14–16^ dental and oral health except oral cancer,^18–24,43^ endocrine nutritional and metabolic disease.^24^ One study found a significant association between passive kretek exposure and oral health.^26^ One study did not find a significant association between smoking kretek cigarettes and skin problems.^43^ Eighteen studies found a significant association between smoking any type of cigarettes and the risk of diseases of the blood and circulatory system,^27–29,31,34,35^ respiratory disease,^33^ mental health problems,^38,44^ eye disease,^40^ diseases of the nervous system,^41^ and oral health problems.^42^ Six studies looked for associations between any cigarette smoking and hypertension stages,^30^ coronary heart disease with single-, double-, or triple-vessel disease,^32^ asthma,^36^ tuberculosis,^37^ depression,^25^ and overweight^39^ and found no association.

Of those reporting significant associations between smoking and health outcomes, most reported the *p*-value only (14 studies),^15,16,19,22,26,27,31,33,35,37–39,41,43^ four studies provided effect size only,^20,23,29,42^ four studies provided a correlation coefficient and *p*-value,^25,32,34,36^ one study provided mean difference and *p*-value,^24^ two studies provided an odds ratio (OR), confidence interval [CI], and *p*-value,^17,40^ one study provided a CI and *p*-value,^30^ one study provided an OR only,^44^ one study provided an OR and *p*-value,^21^ one study provided the relative risk and *p*-value,^18^ one study provided the correlation coefficient only,^28^ and two studies provided the OR and CI.^13,14^

### Studies Investigating Kretek Use and Any Type of Cigarette Use by Indonesian Smokers

#### Cancer

Amtha et al.^13^ found that among 81 cases of oral cancer and 162 controls (nonoral cancer), in an Indonesian hospital setting, after adjusting for alcohol consumption, betel chewing, and dietary pattern, smokers of any type of cigarettes had twice the risk (OR 2.08, 95% CI 1.01–4.43) of having oral cancer when compared with nonsmokers. Kretek smokers were found to have almost double the risk of oral cancer (OR 1.91, 95% CI 0.98–3.95) compared with smokers of other types of cigarettes.

#### Cardiovascular Disease

Of the six studies that looked at associations with cardiovascular disease, all found positive associations. Wasis et al.^14^ found that among 109 900 participants aged 45+ in the Indonesia Basic Health Survey 2007, after adjusting for the population’s residence area, urban–rural, age, level of education and level of expenditure, mixed smokers (kretek and regular cigarettes) had higher risk of cardiovascular disease (OR 1.37, 95% CI 1.25–1.49) compared with nonsmokers. Non-kretek smokers had a slightly higher risk of cardiovascular disease (OR 1.16, 95% CI 1.06–1.27) compared with nonsmokers, as did kretek smokers (OR 1.09, 95% CI 1.02–1.117).

Afriyanti et al.^15^ found that among 47 kretek smokers and 23 regular cigarette smokers in a hospital setting, kretek cigarette smokers had an increased risk of coronary heart disease (*p* = .0001).

Wagiu et al.^16^ reported that among 31 kretek smoker participants with myocardial infarction and 31 without myocardial infarction in a hospital setting, a higher level of cigarette consumption increased the risk of myocardial infarction (*p* = .001).

#### Respiratory Disease

Suharmiati et al.^17^ found that among 15 245 participants in the 2007 Basic Health Survey, after adjusting for confounding factors (age, sex, education, and occupation), kretek cigarette smokers had a higher risk of asthma (OR 1.3, *p* < .001) than non-kretek cigarette smokers.

#### Dental and Oral Health

Soetiarto et al.^18^ found that among 1160 male kretek smokers, smoking 7–12 kretek cigarettes a day increased the risk of having dental decay (2.66, *p* < .0001), and smoking 13–18 kretek cigarettes daily increased the risk threefold (OR 3.19, *p* < .0001), as did smoking over 18 kretek cigarettes a day (OR 2.96, *p* < .0001) when compared with those smoking 0–6 kretek cigarettes a day.

Simamora et al.^19^ found that among 37 kretek smokers and 37 nonsmokers, kretek smokers’ bitter taste sensitivity was higher than that of nonsmokers (*p* = .001). There were no significant differences between sweet (*p* = .39), sour (*p* = .402), and salty (*p* = .07) taste buds’ receptors between kretek smokers and nonsmokers.

Siwi et al.^20^ found that among 94 male smoking miners from Ratatotok, Indonesia, the majority of participants who suffered nicotine stomatitis smoked regular cigarettes, had been smokers for more than 20 years and smoked more than 20 cigarettes daily. One kretek smoker had nicotine stomatitis, whereas 21 (22.3%) of mixed kretek and regular cigarette smokers had nicotine stomatitis.

Nelis et al.^21^ found that among 80 male smokers (40 kretek and 40 regular cigarette smokers), kretek smokers had a five times increased risk (OR 5.174, *p* = .006) of periodontal disease when compared with regular cigarettes smokers.

Kaur et al.^22^ found that among 68 males, 24 regular cigarette smokers and 44 kretek cigarette smokers, smoking kretek increased the risk of having a coated tongue (*p* = .0001) when compared with regular cigarette smokers.

Djokja et al.^23^ found that among 80 male smokers, the majority of those smoking mixed cigarettes (kretek and regular cigarettes) had oral mucosal lesion (92.2%), and the majority of regular cigarette smokers had oral mucosal lesion (81.25%).

#### Endocrine Nutritional and Metabolic Diseases

Khaira et al.^24^ found that among 103 fishermen, smoking kretek cigarettes with a higher nicotine level (>1.5 mg/cigarette) increased the risk of higher waist–hip ratio (WHR) (mean 0.9 ± 0.05) when compared with smoking kretek cigarettes with lower nicotine level (<1.1 mg/cigs) (0.86 ± 0.05) *p* = .025.

#### Skin Problems

Prasetya et al.^43^ found that among 15 regular cigarette smokers, 6 kretek smokers, and 13 mixed kretek and regular smokers, there was no significant relationship between smoking status (*p* = .43), duration and history of smoking (p=0.25), and types of cigarettes consumed (kretek vs non-kretek) (*p* = .32) and face hyperpigmentation.

### Studies Investigating the Association Between Passive Kretek Smoke Exposure and Health Outcomes

#### Melanin Pigmentation of the Gingiva

Setiadhi et al.^26^ found that among 91 children who were nonsmokers (3–12 years), 31 children had fathers who were smokers of any type of cigarettes, 30 had both a mother and father who smoked, and 30 had a nonsmoking father and mother. Children with a smoking father and mother had an increased risk of pigmentation at the labial gingiva (*p* < .001 and *p* < .0001, respectively). Children with a father who smoked kretek cigarettes had increased risk of pigmentation at the labial gingiva (*p* < .001). There was no significant correlation between mother being smoker of kretek cigarettes and increased risk of children’s pigmentation at the labial gingiva (*p* = .063).

### Studies Investigating the Association Between Indonesian Smokers’ Unspecified Cigarette Use and Health Outcomes

#### Cardiovascular Disease

Yashinta et al.^27^ found that among 92 male participants (57 smokers and 35 nonsmokers), smoking any type of cigarettes increased the risk of hypertension (*p* = .0003). Smokers that had smoked for a longer duration had an increased risk of having hypertension (*p* = .017) and smoking nonfilter cigarettes increased the risk of having hypertension (*p* = .017).

Untari et al.^28^ found that among 30 smokers having hypertension, there was a relatively strong correlation between number of cigarettes smoked per day and hypertension (*r*s = .46, *p* = .01).

Narayana et al.^29^ found that among 70 participants (23 smokers and 47 nonsmokers), smoking any type of cigarettes increased the risk of hypertension. Fifty-two percent of smokers had hypertension, and 27.7% nonsmokers had hypertension.

Santosa et al.^30^ found that among 75 (60 females and 15 males) patients in a hospital setting, smoking any type of cigarettes had no significant association with increased risk of stage one hypertension (systolic 130–139 mm Hg, diastolic 80–80 mm Hg) and stage two hypertension (systolic 140 mm Hg or higher and diastolic 90 mm Hg or higher) (*p* = 1.00, OR 0.94, 95% CI 0.11–2.8).

Hikmah et al.^31^ found that among 23 male smokers, a longer duration of smoking increased the risk of having more severe hypertension (*p* = .042).

Ramandika et al.^32^ found that among 153 CHD patients in a hospital setting, smoking cigarettes had no significant effect on the risk of patients having single-, double-, and triple-vessel disease CHD (OR = 1.23, *p* = .56, *r* = .041, 95% CI 0.603–2.5), (OR = 0.88, *p* = .73, *r* = .02, 95% CI 0.45–1.74), and (OR = 0.93, *p* = .83, *r* = .016, 95% CI 0.48–1.79), respectively.

#### Respiratory Disease

Sukmawati et al.^33^ found that among 48 smokers and 48 nonsmokers, mean FVC was lower in smokers (87.02 ± 16.05) than nonsmokers (93.58 ± 14.14), as was FEV1.

Erawati et al.^34^ found that among 40 males (31 smokers and 9 nonsmokers), smoking more cigarettes daily lowered cardiorespiratory endurance (*r* = .497, *p* = .0001).

Rizaldy et al.^35^ found that among 111 high school students, smoking cigarettes lowered cardiorespiratory endurance.

Putra et al.^36^ found that among 49 males and 1 female patient who had asthma in a hospital setting, smoking cigarettes had no significant correlation with increased risk of asthma (*r* = .157, *p* = .275).

Ernawati et al.^37^ found that among 1777 participants (15+), in the Indonesia Basic Health Survey 2010, smoking cigarettes did not increase the risk of tuberculosis (*p* = .489).

#### Mental Health

Wibowo et al.^25^ found that among 33 males university students cigarette smoking had no significant effect on the risk of depression (*p* = 1.00).

Peng et al.^44^ found that among 3061 male and female participants, in the Indonesia Family Life Survey 2007, moderate and heavy smokers increased the risk of depression nearly threefold (OR 2.875) compared with nonsmokers.

Annahri et al.^38^ found that among 108 students cigarette smoking increased the risk of insomnia (*p* = .027) when compared with nonsmokers.

#### Endocrine, Nutritional, and Metabolic Disease

Lestari et al.^39^ found that among 80 participants (12 smokers and 68 nonsmokers) smoking cigarettes had no significant effect on the risk of higher body mass index (*p* > .005) when compared with nonsmokers.

#### Eye Cataracts

Tana et al.^40^ found that among 1223 (566 smokers, 76 ex-smokers, 581 nonsmokers), smoking cigarettes increased the risk of eye cataracts twofold (OR 2.17, 95% CI 1.71–2.75; *p* = .001) when compared with nonsmokers.

#### Nervous System

Noviani et al.^41^ found that among 68 participants (34 with Parkinson’s disease and 34 controls) in a hospital setting, cigarette smoking decreased the risk of Parkinson’s disease (*p* = .002) when compared with nonsmokers.

#### Oral Health

Syawal et al.^42^ found that in a sample of 29 participants there was no association between the level of cigarettes consumed and oral hygiene status.

## Discussion

### Main Findings

This systematic review identified a relatively limited number of studies, and most of these were of poor quality as assessed by the Newcastle–Ottawa score. They were generally cross-sectional, small and lacking justification for sample size, had high potential for selection bias because of lack of data on nonrespondents or those lost to follow up, and missing information about the statistical analysis. Nevertheless, in the context of these limitations, the identified studies generally indicated that kretek cigarettes are associated with increased health risks, both compared with not smoking and in some cases, compared with regular cigarette smoking. We found relatively few studies looking at the health effects of kretek compared with regular cigarettes, most of which were conducted in Indonesia. Studies on the health effects of kretek have looked at a wide range of health outcomes, and most of them showed associations between kretek use and poor health outcomes. Generally, the types of health effects which are associated with kretek use seem to be similar to those for regular cigarettes, such as oral cancer,^15^ cardiovascular disease,^14^ and CHD.^16^ In addition, one study showed a decreased risk of Parkinson’s disease.^41^

### Strengths and Limitations of the Review Methods

This is the first systematic review that has reported the health risks of kretek cigarette smoking compared with not smoking or smoking regular cigarettes. We conducted a thorough literature search using help from an expert librarian to ensure a comprehensive and systematic search strategy. Multiple search strategies were tested to ensure that a comprehensive list of references was identified, we used advance search guidelines to words found in the abstract. The issue of how to deal with studies that did not mention the type of cigarette was problematic, but it is important to recognize that we only included studies which looked at the effects of unspecified cigarettes based in Indonesia, where around 90% of all smokers’ smoke kretek and only around 10% of smoke white cigarettes and hand-rolled cigarette (klembak menyan and tembakau iris).^10,45–47^ Therefore, in the 18 included studies that were conducted in Indonesia, but which do not specifically mention the type of cigarette, it is likely that the majority of tobacco use was kretek. However, we also conducted sensitivity analysis excluding these studies and found no major differences in results. We also included gray literature sources and conducted a focused search to identify studies conducted in Indonesia. We also included studies published in Bahasa Indonesia as well as English to ensure that evidence from Indonesia was captured. A limitation of the review is that one reviewer had to translate written studies in Bahasa for two other reviewers. Furthermore, we may have missed relevant studies written in other languages.

We intended to do meta-analysis if there were studies that had sufficiently similar exposures and outcomes, but the included studies were very heterogeneous, and most were poor quality and had no measures of variability to enable us to undertake meta-analysis.

### Strengths and Limitations of the Included Studies

The included studies covered a wide range of health effects associated with kretek cigarettes. However, a major limitation in this review was that none of the included studies was of good quality. In particular, the included studies were mostly cross-sectional studies, with small samples that were not representative of the general population. Cross-sectional studies measure the prevalence of disease and are thus often called prevalence studies. The measurements of exposure and effect are made at the same time.^48,49^ Consequently, it is not easy to assess the reason for association shown in cross-sectional studies. This, combined with the fact that some of the associations identified were either of borderline statistical significance or the appropriate analysis was not conducted or not presented, limits the extent to which this review can draw conclusions as to the health effects of kretek cigarettes. Cohort studies provide the best information about the causation of disease.^49,50^ However, prospective cohort studies require long follow-up periods and are therefore very expensive and time-consuming,^49^ and there are no obvious sources of data for a retrospective cohort study. To fully understand the health risks of kretek, there is a need for cohort studies and case–control studies.

Furthermore, in all included studies except one where the method was not described, exposure was self-reported. In general, smokers tend to underreport their smoking and therefore smoking could have been under-reported, and dose–response effects may be underestimated. Future studies on the health effects of kreteks should include large representative samples and seek to use biochemical validation.

### Implications

The health risks of kretek cigarettes are not fully understood, and our review demonstrates that good-quality longitudinal studies are needed to properly identify and quantify the risks associated with kretek use. However, despite the limitations of our review methods and the studies included within the review, our findings suggest that kretek are at least as harmful as regular cigarettes. The paucity of evidence does not prevent the need to minimize kretek use, particularly in Indonesia where its use is extremely common.

To date, the FCTC has not been ratified in Indonesia, and the enforcement of existing tobacco control legislation is poor.^3^ Indonesia has minimal smoke-free policy, cessation programs, health warnings, and advertising bans, and cigarettes remain relatively affordable.^51^ Indonesia needs a comprehensive tobacco policy, and it must enforce that policy, compliant with the MPOWER measures proposed by the WHO. Ongoing weaknesses in tobacco control measures in Indonesia are almost certainty contributing to high rates of smoking, particularly among men. Given high rates of use and that they are likely at least as harmful as regular cigarettes, efforts to implement and enforce tobacco control measures in Indonesia should explicitly address kretek use.

The cultural embeddedness of kretek in Indonesian society is likely to make reducing kretek use particularly challenging.^52^ To effectively address kretek use, policymakers require evidence, not just of the health risks that kreteks pose, but also attitudes toward and knowledge about kreteks within the general population. Existing data suggest that Indonesian people understand that smoking cigarettes causes diseases such as lung cancer and heart disease; however, smoking prevalence is still very high, and the number of smokers is not declining.^10^ Research is needed to investigate attitudes and health risk perceptions in relation to both kretek and regular cigarettes in the Indonesian population.

## Conclusions

This systematic review has demonstrated that the existing evidence is insufficient to assess the health risks of kreteks in detail and to fully understand the harms of kretek high-quality longitudinal studies are needed. However, the existing studies suggest that the habitual use of kretek can increase the risk of a range of health problems, including oral diseases, cardiovascular disease, and respiratory disease. Overall, the current evidence base indicates that kretek cigarettes are at least as dangerous as regular cigarettes, and tobacco control efforts in settings where kreteks are commonly used must seek to incorporate measures to reduce kretek use.

## Supplementary Material

A Contributorship Form detailing each author’s specific involvement with this content, as well as any supplementary data, are available online at https://academic.oup.com/ntr.

ntab016_suppl_Supplementary_Appendix

ntab016_suppl_Supplementary_Table_1

ntab016_suppl_Supplementary_Taxonmy_Form
