# Supplementary material for: Health Risks of Kretek Cigarettes: A Systematic Review
Source: Nicotine Tob Res. 2021 Jan 27;23(8):1274–82. doi: 10.1093/ntr/ntab016 (PMC8360627; doi:10.1093/ntr/ntab016)
Supplement: ntab016_suppl_Supplementary_Appendix [file ntab016_suppl_supplementary_appendix.docx]

Appendix 1: PECOS Study design)

| P: Population | Human |
| --- | --- |
| E: Exposure | Did the study look at the following exposure: kretek, specific kretek use, kretek risk factors: smoking-related disease, passive smoking affect (pregnancy, children, etc.)  More specifically, does the study include toxicity of kretek cigarette? |
| C: Comparison, control, or comparator | Current kretek smokers with non-smokers, current kretek smokers with regular cigarettes smokers, non-smokers who are passively exposed to kretek cigarettes, non-smokers who are not exposed or non-smokers exposed to regular cigarettes. |
| O: Outcome (health outcome/ risk/ toxicity) | Long term effects  Short term effects  Benefits for heath of the use and exposure of kretek cigarettes  Physical and mental health or health related outcome |
| S: Study design | We included (if any) current systematic review studies, comparative longitudinal or cohort studies which have assessed the health risk associated with kretek cigarettes. We also included case control and cross-sectional studies.  We excluded animal research. |

Appendix 2: Search Terms

EMBASE and ASSIA: Kretek* OR rokok*OR Merokok* OR (Clove OR Eugnol* adj2 (cigar* OR smok* OR Inhal*)).mp

PubMED : Kretek* OR rokok*OR Merokok* OR (Clove OR Eugnol* adj2 (cigar* OR smok* OR Inhal*))

Scopus: Kretek OR Clove cigar* OR Rokok

Google scholar : “kretek OR Clove cigar OR Rokok*” to fill in the field “with all of the words”, “kretek OR Clove cigarette OR Rokok OR Merokok OR Asap Rokok to fill in the field “with at least one of the words”.

National Library of Indonesia: Rokok, Kretek, Rokok kretek, Merokok, Asap rokok, paparan rokok, rokok Indonesia, Rokok tradisional.

WHO, ASH UK, ASH US, NIH, CDC: Kretek OR Clove cigarette(s)

Medical journals in Indonesia: Rokok, Rokok kretek, Merokok, Asap rokok, paparan rokok, rokok Indonesia, Rokok tradisional.
