# Supplementary material for: Health Risks of Kretek Cigarettes: A Systematic Review
Source: Nicotine Tob Res. 2021 Jan 27;23(8):1274–82. doi: 10.1093/ntr/ntab016 (PMC8360627; doi:10.1093/ntr/ntab016)
Supplement: ntab016_suppl_Supplementary_Table_1 [file ntab016_suppl_supplementary_table_1.docx]

**Supplementary table 1. Main characteristics and findings of included studies**

| Study, year, country | Disease | Sample | Type of cigarette and comparator | Definition of exposure | Main results |
| --- | --- | --- | --- | --- | --- |
| Case-control studies | | | | | |
| Cancer | | | | | |
| Amtha et al, 2014,  Indonesia ^13^ | Oral cancer | Adult hospital patients; Jakarta, Indonesia; n=243 | Kretek vs. other cigarette smokers | No description, but differentiates between non-kretek and kretek smokers | AOR^[[1]](#footnote-1)^ 1.91(95% CI 0.98-3.95) for kretek cigarette vs. other cigarette type smokers. |
| Cross-sectional studies | | | | | |
| Diseases  of blood and circulatory system | | | | | |
| Wasis et al, 2011,  Indonesia ^14^ | CVD | Adult males; Basic Health Survey, 2007, Indonesia; n=100,009 | Mixed (kretek and non-kretek cigarettes) vs. non-smokers, non-kretek smokers vs. non-smokers, kretek smokers vs. non-smokers | Never, former, occasional smokers | AOR 1.37 (95% CI 1.25-1.49) for mixed kretek and non-kretek cigarette smokers vs. non-smokers  AOR 1.16 (95% CI 1.06-1.27) for non-kretek smokers vs. non-smokers  AOR 1.09 (95% CI 1.02-1.17) for kretek smokers vs. non-smokers |
| Yashinta et al, 2015,  Indonesia ^27^ | Hypertension | Adult males; local populations, Indonesia; n=92 | Unspecified cigarettes vs. non-smokers | Duration of smoking <10 years, 10-20, >20.  Number cigarettes consumed daily: <10,10-20, >20 | Significant correlation between smoking and hypertension; r not reported; (p=0.0003). Type of cigarettes and hypertension (p=0.017)  No significant correlation between duration of smoking and hypertension (p=0.071) or the number of cigarettes consumed daily and hypertension. p=0.412) |
| Wagiu et al, 2016, Indonesia ^16^ | Myocardial infarction | Young adult hospital patients; DR Kandao Hospital, Indonesia; n=62 | Kretek vs. kretek based on degree of smoking | Index -Brinkman for the degree of smoking | Significant correlation between the level of smoking and MI (r not reported, p=0.0001) |
| Untari,  2010, Indonesia ^28^ | Hypertension | Adult populations; local villages, Indonesia; n=140 | Unspecified cigarettes. No comparator. | Number cigarettes consumed daily <10; 10-20, >20 | Significant correlation between the number of cigarettes consumed daily and degree of hypertension (r=0.46, p=0.01) |
| Ramandika et al, 2012, Indonesia ^32^ | CHD | Adult hospital patients; Karidao Hospital, Indonesia; n=153 | Unspecified cigarettes vs. non-smokers | No description | No significant correlation between smoking and CHD with:  Single vessel disease (r=0.041, p=0.561)  Double vessel disease (r=0.028, p=0.730)  Triple vessel disease (r=0.016, p=0.839)  No significant correlation between smoking and single, double and triple vessel disease (r=0.048, p=0.841) |
| Narayana et al, 2013, Indonesia ^29^ | Hypertension | Adult populations; local villages, Indonesia; n=70 | Filter and non-filter vs. non-smokers, filter vs. non-filter cigarettes | No description but differentiates between filter and non-filter cigarettes. | 52.2% of smokers (n=12) and 27.2% of non-smokers (n=13) were diagnosed with hypertension. |
| Santosa et al, 2016, Indonesia ^30^ | Hypertension | Adult hospital patients; Kenduri health center, Indonesia; n=75 | Unspecified cigarettes vs. non-smokers | Smoking any type of cigarette | No significant correlation between smoking and hypertension stage I and stage II, (p=1.000; OR =0.942, 95% CI 0.18-5.05) |
| Hikmah et al, 2012, Indonesia ^31^ | Hypertension | Adult males; local population/villages; Indonesia, n=23 | Unspecified cigarettes. No comparator. | Duration of smoking <10 years, 10-20, > 20 | Significant correlation between duration of smoking and degree of hypertension in male smokers (r is not reported, p=0.042) |
| Ratnawulan et al, 2015, Indonesia ^15^ | CHD | Adult hospital patients; DR. Kandao Hospital, Indonesia; n=69 | Kretek vs. regular cigarettes | No description but differentiates between regular cigarettes and kretek | Significant correlation between duration of smoking and CHD (r is not reported, p=0.01), type of cigarette consumed and CHD (p=0.014) type of cigarette consumed and non-CHD (p=0.001). |
| Respiratory diseases | | | | | |
| Sukmawati et al, 2016, Indonesia ^33^ | Pulmonary function (FEF25-75%, FVC1, FEV1, and FEV/FVC) | Adult local populations; Indonesia, n=96 | Unspecified cigarettes vs. non-smokers | No description | Lower mean FVC in smokers (87.02± 19.05) than non-smokers (93.58±14.14) p=0.0036, FEV1 in smokers (82.63±14.47) than non-smokers (94.6±14.43) p<0.0001, FEV1/FVC in smokers (91.77±10.92) than non-smokers (101±9.28) p<0.0001, FEF 25-75% in smokers (66.2±23.58) than non-smokers (96.23±30.43) p<0.0001. |
| Suharmiati et al, 2010, Indonesia ^17^ | Asthma | Population age 10+; Basic Health Research survey 2007, Indonesia; n=15254 | Kretek and regular cigarettes | Daily smokers or occasional smokers | AOR 1.303 for kretek smokers vs. non-smokers, p< 0.001 |
| Rizaldy et al, 2016, Indonesia ^35^ | Cardio- respiratory  Endurance | Young adult school students; Indonesia; n=111 | Unspecified cigarettes vs. non-smokers | Age of starting smoking 7-15 years, duration of smoking 0-30 days, number of cigarettes consumed daily 0->20 | Significant difference between cardiorespiratory endurance of smokers and non-smokers p< 0.001 |
| Erawati et al, 2014, Indonesia^34^ | Cardio- respiratory endurance | Adult university staff; Indonesia; n=48 | Unspecified cigarettes. No comparator. | Index Brinkman for the degree of smoking | Significant correlation between the level of smoking (BI) and cardiorespiratory endurance (r=-0.497, p<0.001) |
| Putra et al, 2012, Indonesia ^36^ | Asthma | Adult hospital patients; DR Djamil hospital, Indonesia; n=228 | Unspecified cigarettes. No comparator. | Index Brinkman for the degree of smoking | No significant correlation between the level of smoking (BI) and degree of asthma exacerbations (r=0.157, p =0.275) |
| Ernawati et al,  2017, Indonesia ^37^ | Tuberculosis of the lungs | Adult population; Basic Health Research survey 2010, Indonesia; n=1777 | Unspecified cigarettes vs. non-smokers | Daily smokers or occasional smokers | No significant correlation between smoking status and tuberculosis (r not reported, p=0.489) |
| Oral disease (except cancer) | | | | | |
| Soetiarto, 1998, Indonesia ^18^ | Dental decay | Adult male bus drivers; Jakarta, Indonesia; n=1160 | Kretek vs. kretek based on number of cigarettes consumed daily | Duration of smoking 6-10 years, 11-15,>15 | RR^[[2]](#footnote-2)^ 2.66 (p<0.001) for smoking 7-12 cigarettes daily vs. 0-6 cigarettes daily.  RR 3.19 (p<0.0001) for smoking more than18 cigarettes daily vs 13-18 cigarettes daily |
| Simamora et al, 2017, Indonesia ^19^ | Taste sensitivity | Adult male pedicab drivers; Medan, Indonesia; n=74 | Kretek vs. non-smokers | Number of cigarettes consumed daily > 5 | Significantly decreased taste perception among smokers compared to non-smokers (p=0.005) between kretek smokers and non-smokers in the sensitivity of taste buds between sweet (p=0.39), sour (p=0.402), and salty (p=0.07) |
| Syawal et al, 2015, Indonesia ^42^ | Gingivitis | Adult males university staff; UNISBA, Indonesia; n=33 | Unspecified cigarettes. No comparator. | No description | Among 18 light smokers: 10 had good oral hygiene, 8 had fair oral hygiene. Among 8 moderate smokers, 6 had fair oral hygiene and 2 had good oral hygiene. Among 3 heavy smokers, 2 had good oral hygiene, and 1 had fair oral hygiene. |
| Siwi et al, 2017, Indonesia ^20^ | Stomatitis nicotine | Adult males gold miners; West Sulawesi, Indonesia; n=94 | Kretek vs regular cigarettes, mixed (kretek and regular cigarettes) vs regular cigarettes | Duration of smoking >1 year | Among 94 respondents, 74 (78.7%) had stomatitis nicotine. Among 64 regular cigarette smokers, 54 (55.3%) had stomatitis nicotine, one kretek smoker had stomatitis nicotine, while 21(22.3%) of mixed kretek and regular cigarette smokers had stomatitis nicotine. The number of people who have nicotine stomatitis in the smokers who smoke less than 10,10-20 and more than 20 cigarettes daily were 7(7.4%),25(26.6%),42(44.5%) respectively. |
| Setiadhi et al, 2011, Indonesia ^26^ | Pigmentation at labial gingiva | Child (aged 0-14) hospital patients; University of Padjajaran hospital, Indonesia; n=90 | Father and mother smoking mixed kreteks and regular cigarettes, father smoking kretek cigarettes, mother smoking kretek cigarettes. | Passive smoking | Significant association between father’s and mother’s smoking and the children’s pigmentation of labial gingiva (p<0.001) and (p<0.001). Significant association between father’s smoking kretek and children’s pigmentation of labial gingiva (p<0.001). No significant association between mother’s smoking kretek and children’s pigmentation of labial gingiva (p<0.001). |
| Nelis et al, 2015 ^21^ | Periodontitis | Adult males; no description, Indonesia; n=80 | Kretek. No comparator. | No description but differentiates between regular cigarettes and kretek | OR 28.66 (CI not reported) for smoking more than 10 years vs. less than 10 years p<0.001  OR 5.174 (CI not reported) for smoking kretek vs. regular cigarettes p=0.0006 |
| Kaur et al, 2013, Indonesia ^22^ | Coated tongue | Adult male University staff; University of Sumatera, Indonesia; n=68 | Kretek vs. regular cigarettes | No description but differentiates between regular cigarettes and kretek | Significant association between kretek vs. regular cigarettes, p<0.001 |
| Djokja et al, 2013, Indonesia ^23^ | Oral mucosal lesion | Adult males; local populations, Indonesia; n=692 | Kretek, regular cigarettes and mixed kretek and regular cigarettes | Number of cigarettes consumed daily >1 and duration of smoking >1 year.  Differentiates between regular cigarettes, kretek and mixed | Rates of oral mucosa lesion cases by the duration of smoking: 1-10 years n=7(8.75%),10-20 years n=10(12.5%) > 20 years of smoking n=51(63.75). Rates of oral mucosal lesion by number of cigs smoked daily: <10 cigs n=11(13.75%), 10-2 cigs n=44(55%), >20 cigs n=13(16.25%). Rates of oral mucosa lesions by type of cigarette: regular n=65(81.25%), kretek n= (0%), mixed regular and kretek n=3(3.375%). |
| Mental health conditions | | | | | |
| Wibowo et al, 2012, Indonesia ^25^ | Depression | Adult male university students; University of Diponegoro, Indonesia; n=33 | Unspecified cigarettes. No comparator. | No description | No significant correlation between smokers vs. non-smokers, p=1 |
| Peng et al, 2015, Indonesia ^44^ | Depression | Adult population; Family life survey 2007, Indonesia; n=3061 | Unspecified cigarettes vs. non-smokers | No description | OR 1.79 (CI not reported) for women smokers vs. men smokers.  OR 2.875 (CI not reported) for heavy smokers vs. non-smokers |
| Annahri et al, 2013, Indonesia ^38^ | Insomnia | Young adult male university students; University of Lambung Mangkurat, Indonesia; n=108 | Unspecified cigarettes vs. non-smokers | Number of cigarettes consumed daily >100, daily or occasional smokers. | Significant association between smoking and insomnia (p=0.027) |
| Endocrine, nutritional and metabolic disease | | | | | |
| Lestari et al, 2017, Indonesia ^39^ | Overweight, obesity, underweight | Adult university staff; Jakarta, Indonesia; n=80 | Unspecified cigarettes vs. non-smokers | No description | No significant association between smoking and BMI (p>0.05) |
| Khaira et al, 2016, Indonesia ^24^ | Waist-hip ratio (WHR) | Adult males fisherman; Padang, Indonesia; n=103 | Kretek with different nicotine content | No description but differentiates between smoking kretek with higher nicotine level vs. lower nicotine level. | Smoking higher nicotine level kretek increased risk of higher WHR (mean 0.9±0.005) as compared to lower nicotine level (<1.1 mg/cigarettes) (0.86±0.05) p=0.025 |
| Other diseases | | | | | |
| Tana et al, 2007, Indonesia ^40^ | Eye ataracts | Adult local populations; Karawang, Indonesia; n=1223 | Unspecified cigarettes vs. non-smokers | Index Brinkman for the degree of smoking | OR 2.17 (95% CI 1.71-2.75) for smokers vs. non-smokers  OR 1.57 (95% CI 1.1-2.2) for heavy smokers vs. non-smoker  OR 4.85 (95% CI 2.0-11.7) for heavy smokers vs. light smokers. |
| Prasetya et al, 2015, Indonesia ^43^ | Hyperpigmentation of the face | Adult male university staff; UNISBA, Indonesia; n=68 | Kretek and regular cigarettes vs. never, former, daily smokers | No description but differentiates between regular cigarettes and kretek | No significant association between smoking daily, smoking less or more than 5 years, ever smoking, and type of cigarettes smoke, p=0.43, p=0.25, p=0.43, and p=0.33, respectively. |
| Noviani et al, 2010, Indonesia ^41^ | Parkinson’s disease | Adult patients; DR Margono, Sidoarjo, Indonesia; n=64 | Unspecified cigarettes vs. Non-smokers | Duration of smoking (0-10 years, 11-20,21-30) | Significant association between smoking and decreased risk of Parkinson’s (p=0.0002) |

1. adjusted odds ratio [↑](#footnote-ref-1)
2. Relative risk [↑](#footnote-ref-2)
